# Protective Effect of *Astragaloside IV* against Cadmium-Induced Damage on Mouse Renal Podocytes (MPC5)

**DOI:** 10.3390/molecules28134897

**Published:** 2023-06-21

**Authors:** Pin Gong, Shan Yue, Fuxiong Shi, Wenjuan Yang, Wenbo Yao, Fuxin Chen, Yuxi Guo

**Affiliations:** 1 School of Food Science and Engineering, Shaanxi University of Science and Technology, Xi’an 710021, China; gongpin@sust.edu.cn (P.G.); 210412126@sust.edu.cn (S.Y.); yangwenjuan@sust.edu.cn (W.Y.); yaowenbo@sust.edu.cn (W.Y.); 2 School of Biological and Pharmaceutical Sciences, Shaanxi University of Science and Technology, Xi’an 710021, China; 3 School of Chemistry and Chemical Engineering, Xi’an University of Science and Technology, Xi’an 710054, China; chenfuxin1981@163.com

**Keywords:** *Astragaloside IV*, cadmium, MPC5, diabetic nephropathy, oxidative stress, mitochondria

## Abstract

In this study, we investigated the protective effect of *Astragaloside IV* (Ast) on mouse podocytes and its possible mechanism of action by constructing a cadmium-induced mouse renal podocytes model. We investigated the effects of cadmium (Cd) toxicity on cell number, morphology, the mitochondrial status of subcellular organelles, protein and gene levels, and the protective effects of Ast by constructing a model of Cd-induced damage to mouse renal podocytes (MPC5) and giving Ast protection at the same time. The results showed that exposure of MPC5 cells to CdCl_2_ culture medium containing 6.25 μM concentration acted with low cell mortality, but the mortality of MPC5 cells increased with the prolongation of cadmium exposure time. Given Ast, the death rate in the low dose group (12.5 μM) was significantly reduced, while the death rate in the medium dose group (25 μM) was extremely significantly reduced. In comparison to the control group, the Cd-exposed group exhibited a significant increase of 166.7% in malondialdehyde (MDA) content and a significant decrease of 17.1% in SOD activity. The mitochondrial membrane potential was also reduced to varying degrees. However, in the Ast-protected group compared to the Cd-exposed group, the MDA content significantly decreased by 20.8%, the SOD activity decreased by 7.14%, and the mitochondrial membrane potential showed a significant increase. Fluorescence staining of mitochondrial membrane potential indicated that Cd exposure caused mitochondrial apoptosis. In the 12-h cadmium-exposed group, the protein expression of Nephrin in mice significantly decreased by 33.4%. However, the expression of the Desmin protein significantly increased by 67.8%, and the expression of the autophagy protein LC3-II significantly increased by 55.5%. Meanwhile, the expression of PINK1, a mitochondrial autophagy pathway protein, was significantly increased in the 12 h and 24 h cadmium exposure groups. The mRNA level of PINK1 was significantly increased, and that of Parkin was decreased in the 48 h cadmium exposure group. Compared to the Cd-exposed group, the Ast group showed more significant improvements in the expression of podocyte structure, functional proteins, and mitochondrial autophagy pathway proteins. The immunological assay of mitochondrial autophagic pathway proteins further indicated that Cd-induced damage to MPC5 cells might be associated with the dysregulation of mitochondrial autophagy.

## 1. Introduction

The pathology of diabetic nephropathy (DN), the main cause of end-stage renal disease (ESRD), may be due to the destruction of the structure and function of the kidney under the high glucose environment, including change the glomerular morphology, degeneration of the renal tubules, and reduction of the creatinine clearance rate [1,2]. According to the International Diabetes Federation (IDF) statistics in 2021, the global number of adults aged 20 to 79 with diabetes was approximately 537 million [1]. It is projected that by 2040, diabetes will become a severe chronic disease affecting the health of around 642 million people. These numbers highlight the growing prevalence and impact of diabetes on a global scale. DN can lead to structural and functional damage to the kidneys, including changes in glomerular morphology, degeneration of renal tubules, and reduced creatinine clearance rate [1,2].

Podocytes play a key role in the pathogenesis of DN [3,4]. Numerous clinical studies have demonstrated the loss of podocyte peduncles in patients with DN, which is closely linked to abnormal glomerular filtration. DN is often accompanied by damage to the renal podocytes, and further studies have shown that most of these damaged podocytes have mitochondrial dysfunction and increased numbers of abnormal mitochondria or abnormal levels of cellular autoregulation [5,6,7,8]. The common basis of podocyte injury is mitochondrial damage and dysfunction and the excessive release of reactive oxygen species (ROS) from damaged mitochondria, which accumulates in cells and tissues, causing protein glycosylation, lipid peroxidation, and nucleic acid damage, further leading to mitochondrial damage and the release of large amounts of mitochondrial pro-apoptotic proteins, resulting in a vicious cycle of damage to podocytes [9,10,11]. This causes oxidative stress lesions in the kidney [12]. Therefore, oxidative stress-induced podocyte damage is an important part of DN podocyte damage [13].

Cadmium, as an environmental and industrial pollutant, has wide-ranging toxicity. The primary sources of cadmium in the environment are industrial and agricultural emissions, which enter the human body through respiration, diet, and drinking water. Due to its slow excretion rate, Cd can accumulate in the liver and kidneys, with a biological half-life of over 10 years. More than 60% of the Cd that enters the body is stored in these organs, leading to renal tubular dysfunction [14,15]. It is now generally accepted that oxidative stress, apoptosis and autophagy and related gene expression, and the interaction of cadmium with sulfhydryl proteins play an important role in the process of cadmium nephrotoxicity [16,17,18]. The vicious cycle of oxidative stress damage caused by the abnormal release of excessive ROS from cells eventually develops into renal dysfunction with tubular reabsorption and disruption of glomerular integrity, while the cadmium-induced increase in lipid peroxidation products in tissues further contributes to organ damage and dysfunction [19,20,21]. Our group has demonstrated that Cd could accelerate the occurrence of DN by breaking the balance of oxidative and antioxidative and also inducing an inflammatory response.

*Astragalus*, a superior food and medicine product, has currently been extensively studied for its biological activity [22,23,24,25,26,27]. *Astragaloside IV* (Ast), the most important active component of *Astragalus*, is often used as a strong oxidant for the purpose of oxidative stress damage due to its ability to effectively scavenge free radicals [28,29,30,31]. Ast has been shown to have anti-diabetic effects and renal-protective effects [32,33,34,35]. However, the specific mechanism of action of Ast in protecting the kidney in DN models is not fully understood in the current study, and further research is needed to elucidate these issues.

In this study, we intend to investigate the protective effect of Ast and its possible mechanism of action by constructing a model of cadmium-induced renal podocyte injury; our data may provide new insight for the development of drug candidates in the clinical therapy of DN.

## 2. Results

### 2.1. Effect of Ast Intervention on Morphological Changes in MPC5

Preliminary screening based on the results of the pre-experiment was carried out at Ast action concentrations of 12.5 μM, 25 μM and 50 μM as low, medium, and high doses, respectively, and cadmium-damaged MPC5 cells were cultured for 24 h as the concentration and action time for the later treatment. The pre-experimental results showed that the mortality rate was significantly lower in the Ast low dose group (*p* < 0.05), in the Ast medium dose group (*p* < 0.01), and no significant difference in the Ast high dose group (50 μM) compared to the CdCl_2_ treated group, probably due to the high concentration of Ast action, which led to severe cell necrosis. Therefore, the experimental concentration of 25 μM Ast was chosen for the subsequent investigation.

**Figure 1:**
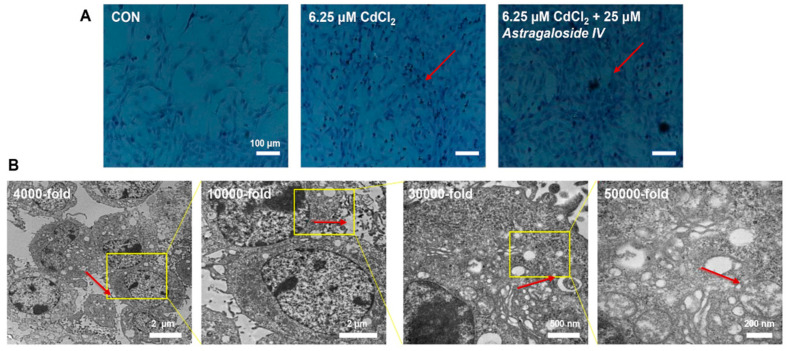
Effect of Ast on morphology (**A**) and TEM ultrastructure (**B**) of cadmium-induced MPC5.

As shown in Figure 1A, MPC5 cells in the CON group maintained their post-differentiation cell state, with intact morphology, well situated, polygonal, and tiled, with obvious and easy wall apposition, and large and intact cytosol; after cadmium intervention with heavy metal, apoptosis was obvious, and cells were wrinkled. After 24 h of cadmium intervention and then 25 μM Ast intervention, the morphology was obviously improved after Ast treatment, which was close to the cell morphology of the CON group. It further indicated that Ast could effectively alleviate the burden of renal MPC5 cells caused by heavy metal cadmium and played a protective role in the normal physiological function of renal MPC5 cells.

The ultrastructure of Ast acting on cadmium-induced MPC5-damaged cells is depicted in Figure 1B. Compared with the cadmium-exposed group, Ast could effectively improve the apoptotic ability of the cells; the number of cells in the light-colored part was less, the overall cell structure was relatively intact, the mitochondrial bilayer membrane structure was intact, there was no serious intercellular adhesion and diffuse fusion of contents, the intercellular matrix was relatively neatly arranged, and there were fewer mitochondrial autophagic vesicles. It indicates that Ast can effectively ameliorate the burden of MPC5 cell function caused by cadmium and play a certain protective role for renal MPC5 cells.

### 2.2. MDA, ROS Content, and SOD Activity after Ast Intervention

**Figure 2:**
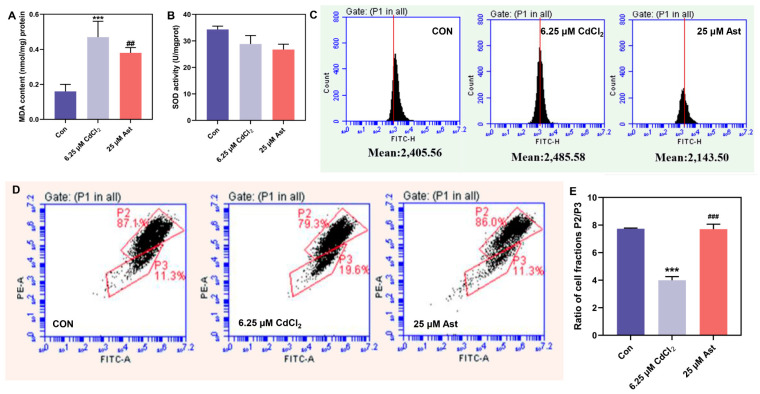
Effect of Ast intervention on antioxidant depolarizing capacity. (**A**) MDA content; (**B**) SOD activity; (**C**) ROS content change in different groups. (**D**,**E**) Degree of depolarization of mitochondrial membrane potential in different groups. Compared with Con group *** *p* < 0.001, compared with 6.25 μM CdCl_2_ group ## *p* < 0.01, ### *p* < 0.001.

As indicated in Figure 2A, the MDA content was significantly increased in the 6.25 μM CdCl_2_ group compared to the CON group (*p* < 0.01), with severe oxidative cellular damage. Compared with the 6.25 μM CdCl_2_ group, the MDA content in the Ast treatment group significantly decreased (*p* < 0.05). This indicates that Ast can effectively improve the degree of oxidative damage caused by cadmium and reduce the accumulation of oxidation products.

As demonstrated in Figure 2B, in the 6.25 μM CdCl_2_ group, the antioxidant level, as indicated by SOD activity, was decreased compared to the CON group. Furthermore, in the Ast group, SOD activity was also decreased compared to the 6.25 μM CdCl_2_ group. This indicates that cadmium exposure led to a decrease in the antioxidant capacity of the podocytes mainly by decreasing the cellular antioxidant level SOD activity, while Ast did not significantly improve the decrease in SOD activity, thus indicating that the potential effect of Ast on cadmium-induced MPC5 damage is not achieved through increasing SOD activity.

The results of ROS levels in different groups (Figure 2C) demonstrated that the relative value of ROS was increased in the 6.25 μM CdCl_2_ group compared to the CON group. The peak of ROS levels was slightly shifted to the right, indicating a potential increase in ROS content. However, the shift was not significant. Compared with the 6.25 μM CdCl_2_ group, the ROS relative value decreased in the Ast-treated group. This indicates that Ast can effectively improve the excessive release of ROS from damaged cells caused by cadmium and maintain it at a relatively balanced level.

### 2.3. Effect of Ast Intervention on Mitochondrial Membrane Potential

The mitochondrial membrane potential flow assay results in different groups of cells are shown in Figure 2D. Compared with CON, the high potential aggregates P2 in the 6.25 μM CdCl_2_ group changed from strong too weak to low potential monomers P3 from weak to strong. The ratio of cell population P2/P3 was significantly lower (*p* < 0.01) (Figure 2E). Compared to the group treated with 6.25 μM CdCl2, the Ast group exhibited an elevation in the high-potential polymer P2 and a reduction in the low-potential monomer P3. Moreover, the ratio of cell population P2/P3 was significantly higher (*p* < 0.01) in the Ast group (Figure 2E). It indicates that Ast can protect mitochondria and improve cellular mitochondrial damage caused by cadmium.

**Figure 3:**
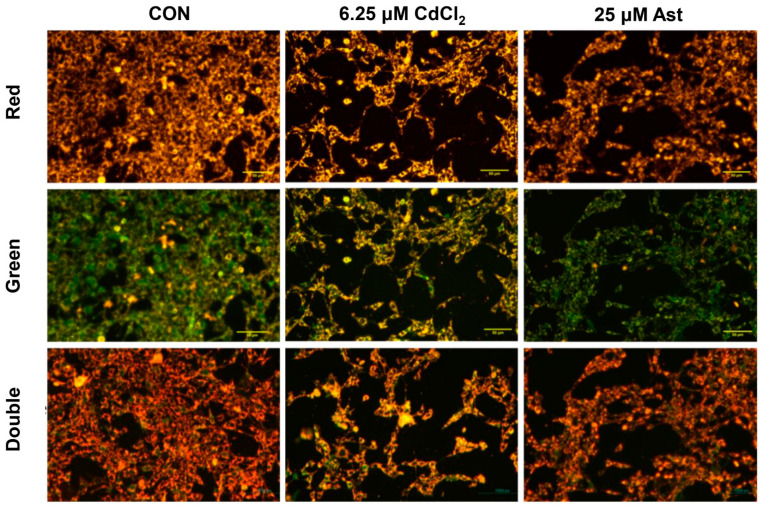
JC-1 to observe the changes in mitochondrial membrane potential in different groups.

Observation of mitochondrial JC-1 staining in different groups of cells (Figure 3) showed that the 6.25 μM CdCl_2_ group had a dramatic decrease in the number of cells, a smaller fluorescence area, and an increase in the merged graph green light area and more mitochondrial low potential monomers compared to the CON group. Compared to the 6.25 μM CdCl_2_ group, the Ast treatment group had a higher number of cells, a significantly larger fluorescence area and more mitochondrial high-potential red fluorescence. It indicates that Ast can improve the damage caused by cadmium by protecting the structure of mitochondria and, thus, the damage caused by cadmium.

### 2.4. Effect of Ast Intervention on the mRNA Expression Levels of PINK1/Parkin Pathway in Cadmium Treated MPC5 Cells at Different Times

**Figure 4:**
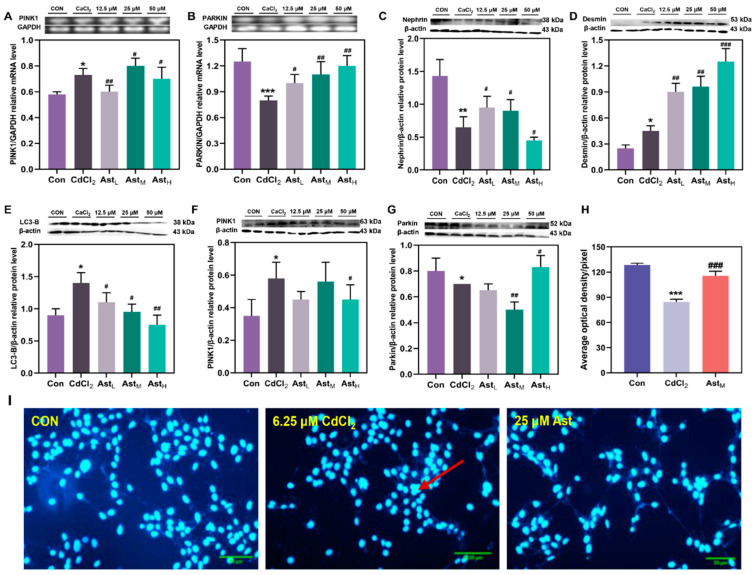
PINK1/Parkin mRNA and protein expression and immunofluorescence chemical detection after Ast intervention. (**A**,**B**). mRNA levels change of PINK1/Parkin pathway in MPC5 exposed to CdCl_2_; (**C**,**D**). Structural and the functional protein Nephrinand Desmin content changes of MPC5 exposed to CdCl_2_; (**E**). Cell autophagy protein LC3-II content changes; (**F**,**G**) Protein content change of in PINK1/Parkin pathway in MPC5 exposed to CdCl_2_; (**H**,**I**) Changes of immunofluorescence intensity in PINK1/Parkin pathway observed by DAPI staining at MPC5 exposed to CdCl_2_). Compared with CON group * *p* < 0.05, ** *p* < 0.01, *** *p* < 0.001, compared with CdCl_2_ group # *p* < 0.05, ## *p* < 0.01, ### *p* < 0.001.

As demonstrated in Figure 4A, the mRNA levels of PINK1 were significantly increased in the CdCl_2_ group compared to the CON group (*p* < 0.05). Compared with the CdCl_2_ group, the transcript levels of PINK1 were significantly up-regulated in the Ast low-dose group (*p* < 0.01) and in the medium and high-dose groups (*p* < 0.05). It indicates that Ast can improve the changes of mRNA levels of PINK1 of the mitochondrial autophagic pathway caused by cadmium and maintain it at a normal level. As depicted in Figure 4B, the mRNA level of Parkin was significantly up-regulated in the CdCl_2_ group compared to the CON group (*p* < 0.01). The mRNA levels of Parkin were significantly up-regulated in the Ast all-dose group compared to the CdCl_2_ group. This indicates that Ast can ameliorate the changes in mRNA levels of Parkin, a mitochondrial autophagic pathway caused by cadmium, and maintain it at normal levels.

### 2.5. Effect of Ast Intervention on the Protein Expression Levels of PINK1/Parkin Pathway in Cadmium-Treated MPC5 Cells at Different Times

Analysis of Nephrin protein expression in MPC5 cells in each group showed (Figure 4C) that Nephrin protein expression was significantly more significantly reduced by 33.4% in the cadmium-exposed group compared to the CON group (*p* < 0.01). Compared to CdCl_2_, the protein expression was significantly up-regulated by 38.61% and 38.02% in the low and medium dose groups of Ast, respectively (*p* < 0.05). This indicates that Ast can improve the expression of functional protein content in MPC5 cells caused by Cd to maintain it at normal levels. The expression analysis of the Desmin protein in MPC5 cells is shown in Figure 4D. Desmin protein expression was significantly up-regulated in the CdCl_2_ group compared to the CON group (*p* < 0.05). Desmin protein levels were significantly higher in the Ast all dose groups compared to the CdCl_2_ group (*p* < 0.05).

As illustrated in Figure 4E, LC3B protein expression was significantly up-regulated in the CdCl_2_ group compared to the CON group (*p* < 0.05). Protein expression was down-regulated by 17.68% (*p* < 0.05), 33.32% (*p* < 0.05), and 34.13% (*p* < 0.05) in the Ast low, medium, and high dose groups, respectively, compared to the CdCl_2_ group. As shown in Figure 4F, analysis of mitochondrial autophagic pathway protein PINK1 expression showed that protein expression was significantly upregulated in the CdCl_2_ group compared to the CON group (*p* < 0.05). Protein expression was down-regulated by 29.31% (*p* < 0.05) and 29.07% (*p* < 0.05) in the Ast low-dose and high-dose groups, respectively, compared to the CdCl_2_ group. As demonstrated in Figure 4G, analysis of the expression of Parkin, a protein downstream of the mitochondrial autophagic pathway, showed significant downregulation of protein expression in the CdCl_2_ group compared to the N group (*p* < 0.05). Compared with the CdCl_2_ group, the Ast medium dose group was significantly down-regulated by 28.34% (*p* < 0.05). This indicates that Ast can effectively improve the changes of mitochondrial autophagy protein content in MPC5 cells caused by cadmium and maintain it at a normal level.

### 2.6. Effect of Ast Intervention on Immunofluorescence Assay in Cadmium-Treated MPC5 Cells at Different Times

Immunofluorescence observation of PINK1/Parkin proteins of the mitochondrial autophagic pathway in different groups of cells was performed by DAPI staining, as shown in Figure 4H,I. Compared with the CON group, the cells in the 6.25 μM CdCl_2_ group were severely damaged, with a significantly reduced fluorescence area (*p* < 0.01), adhesions between damaged cells, and diffuse fusion of contents, and therefore, lamellar, clumped, and drawn fluorescence in the protein immunodetection; compared with the 6.25 μM CdCl_2_ group, the cell status of the Ast treatment group was close to that of the CON group, with significantly higher fluorescence intensity (*p* < 0.01). This indicates that Ast can improve the changes of mitochondrial autophagic pathway proteins caused by cadmium and make them normalize.

## 3. Discussion

As a poorly proliferating but highly differentiated and specific cell, the different functions of the podocyte depend on its complex three-part structure, namely the cell body, the primary protrusion, and the peduncle [36,37]. The function of the “finger-like interlocking” peduncle is multi-functional and is reflected in the fact that the peduncle can effectively help the cell to grow against the wall so that early cell damage can be manifested as shrinkage and rounding of the cell due to the loss of the peduncle and the poor ability to adhere to the wall, followed by shrinking of the cell body, severe apoptosis and reduced proliferation [2,38,39].

After exposing MPC5 cells to cadmium, a significant reduction in cell proliferation capacity was observed. The cell mortality assay results indicated an increase in cadmium-induced cell death that was both dose-dependent and time-dependent. The morphological changes of the cells were observed that the cell structure was altered under cadmium exposure, with shrinking of the cytosol, retraction of the peduncle, pseudocyst formation, and vacuolization after 24 h of cadmium intervention; further TEM results showed that the cell integrity was disrupted under cadmium exposure and apoptosis became more severe with longer exposure time. The mitochondrial structure of the subcellular organelle was damaged and dysfunctional, unable to effectively scavenge oxygen radicals, and the number of damaged mitochondria increased and could not be effectively scavenged [40,41,42]. The excessive release of ROS from damaged mitochondria led to oxidative damage, glomerular structural lesions causing dysfunction, increased proteinuria, progressive development of glomerulosclerosis, and eventual loss of renal function.

Early DN lesions are accompanied by podocyte apoptosis [43,44]. Studies on the relationship between podocyte structure and function and the development of proteinuria in diabetic nephropathy have shown that Nephrin, as a transmembrane protein and signaling receptor molecule, apart from participating in cellular signaling, its most important role is to maintain the normal morphology and function of the podocyte together with the cytoskeleton [45]. Exposure to cadmium disrupts the linkage structure between the cytoskeleton and Nephrin protein. This disruption leads to a decrease in the escape of Nephrin protein, and its expression is reduced in a time-dependent manner following cadmium exposure [46,47].

The present study revealed that the expression of the structural protein Desmin increased in a time-dependent manner when exposed to cadmium, indicating that MPC5 cells were damaged [48,49]. LC3 is a marker of cellular autophagy, and the amount of LC3-II was positively correlated with the degree of autophagy occurring [50]. The present study showed that the expression of LC3-II, a functional protein of autophagy, increased in a time-dependent manner after cadmium treatment, and the degree of autophagy was severe. In conclusion, MPC5 cells, as the most vulnerable component of the glomerular structure, underwent abnormal structural and functional changes under contamination with the heavy toxic substance cadmium, affecting their normal level of autophagy regulation and apoptosis. As highly differentiated cells with poor proliferative capacity, maintaining their numerical homeostasis is crucial in the protection of glomerular filtration membrane integrity.

**Figure 5:**
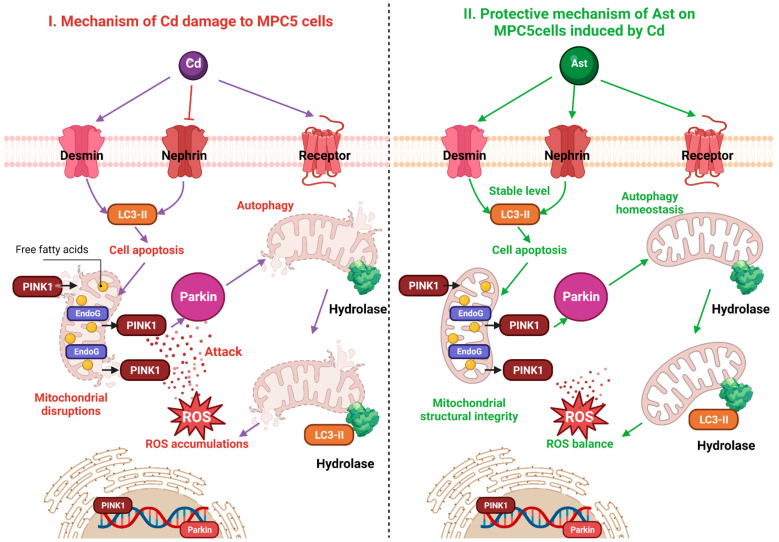
Mechanism of cadmium-induced damage to MPC5 cells and the mechanism of Ast protection.

The mechanism of cadmium-induced damage to MPC5 cells and Ast protection are shown in Figure 5. The mechanism of cadmium-induced MPC5 cell pathogenesis is mainly caused by an imbalance in the number and quality of mitochondria and excessive release of ROS due to abnormal mitochondrial clearance capacity, i.e., impaired mitochondrial autophagy pathway, in MPC5 cells damaged by cadmium (Figure 5, left). The protective mechanism of Ast treatment is to maintain the homeostasis of mitochondrial quantity and quality by medicating PINK1/Parkin pathway, thereby reducing the release of ROS, elevating the antioxidant levels, and finally, protecting against the toxicity of cadmium on renal podocytes. (Figure 5 right).

## 4. Materials and Methods

### 4.1. Cell Line

The immortalized mouse podocyte cells (Mouse Podocyte Cells 5, MPC5) used in the experiments were a gift from Northwest University Shaanxi Province.

### 4.2. Materials, Chemicals, and Antibody

Cadmium chloride (analytical purity) was purchased from Tianjin Fuchen Chemical Reagent Factory. RPMI 1640 medium was purchased from Corning, Oneonta, NY, USA. Fetal bovine serum, penicillin–streptomycin, trypsin–EDTA digest, recombinant γ-interferon were purchased from Bioengineering (Shanghai) Co. (Shanghai, China). Taipan Blue staining solution was purchased from Beijing Solabao Biotechnology Co. (Beijing, China). Immunostaining permeation solution (Triton X-100) was purchased from Beyoncé Biotechnology Co. (Shanghai, China).

Anti-Nephrin antibodies, Anti-LC3-II antibodies, and Anti-Desmin antibodies were purchased from Abcam. Rabbit anti-PINK1 polyclonal antibody, Parkin Antibody, was purchased from Bioengineering (Shanghai) Co.

### 4.3. Establishment of Cell Models and Cell Experiments

By inducing Cd exposure in MPC5 cells and screening culture conditions, the optimal modeling conditions were determined as follows: exposing MPC5 cells to a culture medium containing a concentration of 6.25 μM CdCl_2_ for 24 h to establish a cadmium-induced injury model of MPC5 cells. Different doses of Ast (12.5, 25, 60 μM) were added to cadmium-injured MPC5 cells and treated for 24 h. The protective group was obtained for subsequent experiments.

### 4.4. Influence of Oxidative Stress on MPC5 Injuries

#### 4.4.1. Determination of Cellular Malondialdehyde (MDA) Levels and Superoxide Dismutase (SOD) Activity

Cells were treated with cadmium at different time periods and then lysed on ice for 30 min with IP cell lysis solution (0.1 mL of lysis solution was used per 106 cells). The cell lysate was fully collected with a cell scraper, centrifuged at 1600× *g* for 10 min, and the supernatant was taken for subsequent assays. The protein concentration was determined using the BCA protein concentration assay kit (Nanjing Jiancheng Institute of Biological Engineering, Nanjing, Jiangsu, China). The MDA content and SOD activity were determined using the TBA and NBT methods, respectively [51].

#### 4.4.2. DCFH-DA Staining for Intracellular Total ROS Levels

2′7′-Dichorofluorescin diacetate (DCHF-DA) powder was prepared to a final concentration of 10 μmol/L, and serum-free RPMI1640 medium was used as the diluent [52]. The cells were incubated in an incubator at 37 °C for 30 min. The original medium was removed, and the cells were washed again with PBS buffer 2–3 times. Add 1 mL of flow cytometry digestive enzyme Acc and digest for 3 min in 37 °C incubator. After the cells become round, add 4 times more culture medium to terminate the digestion, collect the cell solution in a 15 mL centrifuge tube, 1000 r/min, centrifuge for 3 min, discard the supernatant, add 1 mL PBS buffer to resuspend the cells, filter with 300 mesh nylon filter, put the liquid on the flow cytometer as soon as possible detect the fluorescence value. Set the following parameters for operation: excitation wavelength of 488 nm and emission wavelength of 525 nm.

### 4.5. Characterization of Mitochondrial Membrane Potential

#### 4.5.1. Detecting the Degree of Mitochondrial Membrane Potential Depolarization

The degree of mitochondrial membrane potential depolarization was measured by flow cytometry [53,54]. Briefly, 5,5′,6,6′-Tetrachloro-1,1′,3,3′-tetraethyl-imidacarbocyanine iodide (JC-1) red powder was the cells were dissolved in DMSO and diluted in serum-free RPMI1640 medium to a final concentration of 10 μg/mL. Wash the cells 2–3 times with PBS, collect the cells by flow special enzyme Acc digestion, centrifuge at 1000 r/min for 3 min, remove the supernatant, add 1 mL PBS to resuspend the cells, take 100 μL of cell suspension into a 2 mL centrifuge tube, add 400 μL PBS to dilute the cell suspension, pass through a 300-mesh nylon mesh, filtrate onto a flow cytometer for operation.

#### 4.5.2. Measurement of Mitochondrial Membrane Potential

The mitochondrial membrane potential of living cells was measured by JC-1 staining [55]. In brief, mature MPC5 cells were subjected to intervention with CdCl_2_ for 0 h, 12 h, 24 h, and 48 h. Following the treatment, the original culture medium was aspirated, the cells were washed 2–3 times with PBS buffer, the above diluted JC-1 staining solution was added, incubated in an incubator at 37 °C for 30 min, the original staining solution was removed, the cells were washed 2–3 times with PBS buffer, 1 mL of PBS buffer was added The cells were observed under an inverted fluorescent microscope, with fluorescence at excitation light of B450–490 nm, and G510–560 nm, and the ratio of red to green fluorescence was observed and photographed [56].

### 4.6. Quantitative Real-Time Polymerase Chain Reaction (qRT-PCR) Analysis

The mRNA expression of the PINK1/Parkin was determined by qRT-PCR [57,58,59]. The purity was measured with the aid of a nucleic acid quantification instrument. The solution was dispensed in enzyme-free tubes and stored at −80 °C. The primer design process was mainly carried out according to Table S1. The first strand of cDNA was retrotranscribed, and the expression of each gene was quantified in real time and normalized to the expression of 3-phosphoglycerate dehydrogenase (GAPDH) in the same sample using a one-step qRT-PCR kit from Sangon Biotech(Shanghai, China). The relative expression was measured using 2^−ΔΔCt^ method.

### 4.7. Western Blot Analysis

Total protein from the cells was extracted using radioimmunoprecipitation assay (RIPA) lysis buffer according to the instructions provided in the kit (Xi’an Haite Biotechnology Co., Xi’an, China) [60,61]. Briefly, the membranes were then washed three times with TBST and incubated for 1 h at room temperature with the appropriate secondary antibody conjugated to horseradish peroxidase (HRP). Immunoreactive bands were observed using an enhanced chemiluminescence (ECL) kit (Millipore Co., Billerica, MA, USA), with GAPDH as an internal control.

### 4.8. Statistical Analysis

The results are expressed as mean ± SD, and differences between groups were assessed by one-way analysis of variance (ANOVA) using the LSD test. Differences were considered statistically significant at *p* < 0.05. Statistical analyses were performed using SPSS (IBM SPSS-Statistics, Wuxi, China).

## 5. Conclusions

In this study, cadmium chloride was used as an induction drug, and immortalized mouse podocytes MPC5 were used as the target. The aim was to construct a cadmium-induced DN cell model and to investigate the mechanism of cadmium-induced damage to MPC5 cells and the effect of Ast on delaying the progression of cadmium-induced oxidative damage to MPC5 cells. Our data will provide new insight into the clinical application of Ast as a drug candidate in the therapy of DN.

## Data Availability

Not applicable.

## Supplementary Materials

The following supporting information can be downloaded at: https://www.mdpi.com/article/10.3390/molecules28134897/s1, Table S1: Primer Sequence of DNA.
