# Supplementary material for: Protective Effect of Astragaloside IV against Cadmium-Induced Damage on Mouse Renal Podocytes (MPC5)
Source: Molecules. 2023 Jun 21;28(13):4897. doi: 10.3390/molecules28134897 (PMC10343813; doi:10.3390/molecules28134897)
Supplement: Supplementary file 1 [file molecules-28-04897-s001.zip › molecules-2430985-supplementary.pdf]

Supplementary materials

Table S1. Primer Sequence of DNA

Gene	Primer Sequence (5′-3′)	TM value
PINK1-F	5′-ATCAGTAGCATCTAGCATAC-3′	47.9
PINK1-R	5′-GATCACTGATCAGATCTATCC-3′	48.8
Parkin-F	5′-CATGATCAGTTCATGATCACT-3′	49.1
Parkin-R	5′-ATCTAGCATTAGCTAACTC-3′	44.6
GAPDH-F	5′-TCAAGAAGGTGGTGAAGCAG-3′	58.3
GAPDH-R	5′-AGGTGGAAGAATGGGAGTTG-3′	55.7
